# Enhancing medical imaging education: integrating computing technologies, digital image processing and artificial intelligence

**DOI:** 10.1002/jmrs.837

**Published:** 2024-11-07

**Authors:** Sibusiso Mdletshe, Alan Wang

**Affiliations:** ^1^ Department of Anatomy and Medical Imaging, Faculty of Medical and Health Sciences The University of Auckland Auckland New Zealand; ^2^ Auckland Bioengineering Institute The University of Auckland Auckland New Zealand; ^3^ Medical Imaging Research centre, Faculty of Medical and Health Sciences The University of Auckland Auckland New Zealand; ^4^ Centre for Co‐Created Ageing Research The University of Auckland Auckland New Zealand; ^5^ Centre for Brain Research The University of Auckland Auckland New Zealand

## Abstract

The rapid advancement of technology has brought significant changes to various fields, including medical imaging (MI). This discussion paper explores the integration of computing technologies (e.g. Python and MATLAB), digital image processing (e.g. image enhancement, segmentation and three‐dimensional reconstruction) and artificial intelligence (AI) into the undergraduate MI curriculum. By examining current educational practices, gaps and limitations that hinder the development of future‐ready MI professionals are identified. A comprehensive curriculum framework is proposed, incorporating essential computational skills, advanced image processing techniques and state‐of‐the‐art AI tools, such as large language models like ChatGPT. The proposed curriculum framework aims to improve the quality of MI education significantly and better equip students for future professional practice and challenges while enhancing diagnostic accuracy, improving workflow efficiency and preparing students for the evolving demands of the MI field.

## Graphical abstract

This paper has proposed a comprehensive curriculum framework that integrates computing technologies, digital image processing and artificial intelligence into medical imaging education, aiming to prepare future professionals to navigate and innovate within this dynamic landscape.

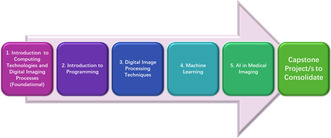

## Introduction

The recent surge in computational power and the development of sophisticated algorithms necessitate the integration of more advanced computational and digital imaging processing skills into the medical imaging (MI) educational framework.^1^, ^2^ Computing technologies, including programming, data analysis and machine learning (ML) skills are becoming increasingly vital in the MI field.^3^, ^4^ These skills enable MI professionals to develop and utilise sophisticated imaging algorithms, manage and analyse large datasets and optimise imaging protocols to enhance diagnostic accuracy and efficiency. Digital imaging processing techniques, such as image enhancement, segmentation, registration and three‐dimensional reconstruction, play a crucial role in refining image quality and providing detailed insights into anatomical structures and pathological conditions.

In addition, the MI practice includes several skills that require advanced computer skills, that is organisation, quality control, communication, image analysis, digital imaging and engaging with Picture Archiving and Communications System (PACS) and Radiology Information System (RIS).^5^ Notably, artificial intelligence (AI) algorithms, particularly those based on deep learning (DL), have demonstrated remarkable success in image recognition and analysis tasks, often surpassing human performance in various diagnostic applications.^6^ Further, AI can assist in maximising image quality by reducing noise and artefacts and can also improve the early detection of various diseases.^3^ The integration of large language models, such as ChatGPT, offers additional opportunities to enhance education through interactive learning tools and personalised tutoring, providing students with a more engaging and effective learning experience.^7^

Several publications have highlighted the role of MI technologists (MITs) in the AI era.^8^, ^9^ In addition, some professional societies have adopted or embedded AI education and training within their guidelines/career frameworks.^10^ The joint statement by the International Society of Radiographers and Radiological Technologists (ISRRT) and the European Federation of Radiographer Societies (EFRS) highlights the need for change in practice for MITs that is underpinned by appropriate education and training that involves the current practitioners and the future practitioners, and high‐quality research evidence.^11^ This change in practice could enable several benefits, including enhanced dose reduction and image optimisation workflows; diagnostic accuracy and efficiency; evidence‐based clinical practice and clinical decision‐making; patient pathways and outcomes; and opportunities for involvement in piloting and research of algorithms before their use in the clinical sphere.^11^, ^12^, ^13^

Despite the clear benefits of integrating these advanced technologies into the MI curriculum, many educational programmes have been slow to adapt. Nonetheless, there is a pressing need to modernise MI education better to prepare students for the evolving demands of the profession.^14^, ^15^

This paper aims to address this gap by proposing a comprehensive curriculum framework that incorporates computing technologies, digital imaging processing and AI that could prepare students better for the challenges and opportunities of the modern MI landscape. In the following sections, an overview of the current state of MI education is provided, followed by a discussion of the importance of integrating advanced technologies and a presentation of an envisaged curriculum framework for MI education. Additionally, the potential challenges and considerations in implementing this curriculum are explored while offering strategies for overcoming these barriers.

## Current State of MI Education

A review of selected MI curricula was done to understand the current state of MI education. The universities/societies/regulatory bodies were selected to include a variety of contexts (including the New Zealand context), and the curriculum needed to be available online. This review was done as a desktop review and it showed that MI education traditionally centers on providing technological, social and biological knowledge that prepares the students for clinical practice and registration with the relevant regulatory body.^10^, ^16^, ^17^, ^18^, ^19^, ^20^, ^21^ The core curriculum typically includes courses in anatomy, physiology, radiographic positioning, radiation protection and basic pathology, complemented by practical training in clinical settings and/or simulation facilities.

While these foundational elements are crucial, the current educational framework often lacks the integration of advanced computational and digital imaging processing skills that are increasingly essential in today's MI field.^22^, ^23^ Further, the competence standards that are prescribed by the regulatory bodies generally lack reference to computing technologies, digital image processing and AI.^10^, ^24^, ^25^ A recent study by Stogiannos et al.^9^ highlights that among the reasons for not incorporating AI in curricula include lack of knowledge/expertise among educators, guidance to develop curriculum, time to allocate resources, funding and infrastructure, engagement from colleagues, interest from senior management and approval from senior management.

The current state of MI education highlights the need for a more comprehensive and integrated approach that incorporates advanced computational skills, digital image processing techniques and AI tools to modernise the curriculum. Several institutions have effectively integrated computing technologies, digital image processing and AI into their MI curricula. For instance, the University of Sydney has introduced the ‘AI in MI’ that explores the state of the art and prospects for AI applications in MI.^26^ Similarly, the City University of London has implemented a professional development course ‘Introduction to AI for Radiographers’.^27^, ^28^ This course equips attendees with an understanding of the potential impact of AI on MI practice and prepares them for future practice. In addition, University College London (UCL) has established a Centre for Medical Image Computing which, in addition to research, provides education (e.g. Medical Image Computing Summer School).^29^

## Proposed Curriculum Framework for MI Education

**Figure 1:**
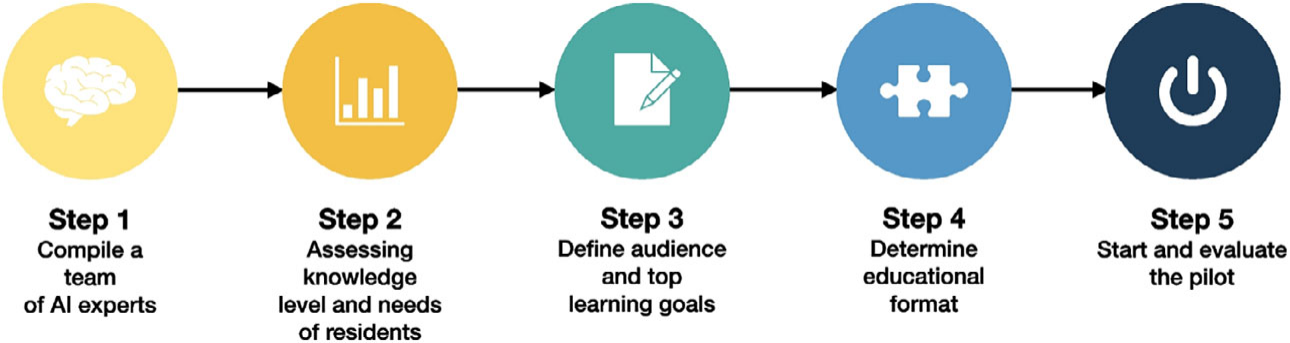
A five‐step framework to develop and implement an AI curriculum into an existing radiology residency program.^3^ AI, artificial intelligence. *Reproduced without amendments from van Kooten* et al. *under Creative Commons licence CC‐BY 4.0. DOI:* https://doi.org/10.1186/s13244‐023‐01595‐3.

To address the evolving demands of the MI field and capitalise on the benefits of integrating computing technologies, digital image processing and AI, a comprehensive curriculum framework is proposed. The proposed framework also considers the recent work by van Kooten et al.,^3^ which presents a framework for integrating AI training into radiology residency programmes (Fig. 1).

**Figure 2:**
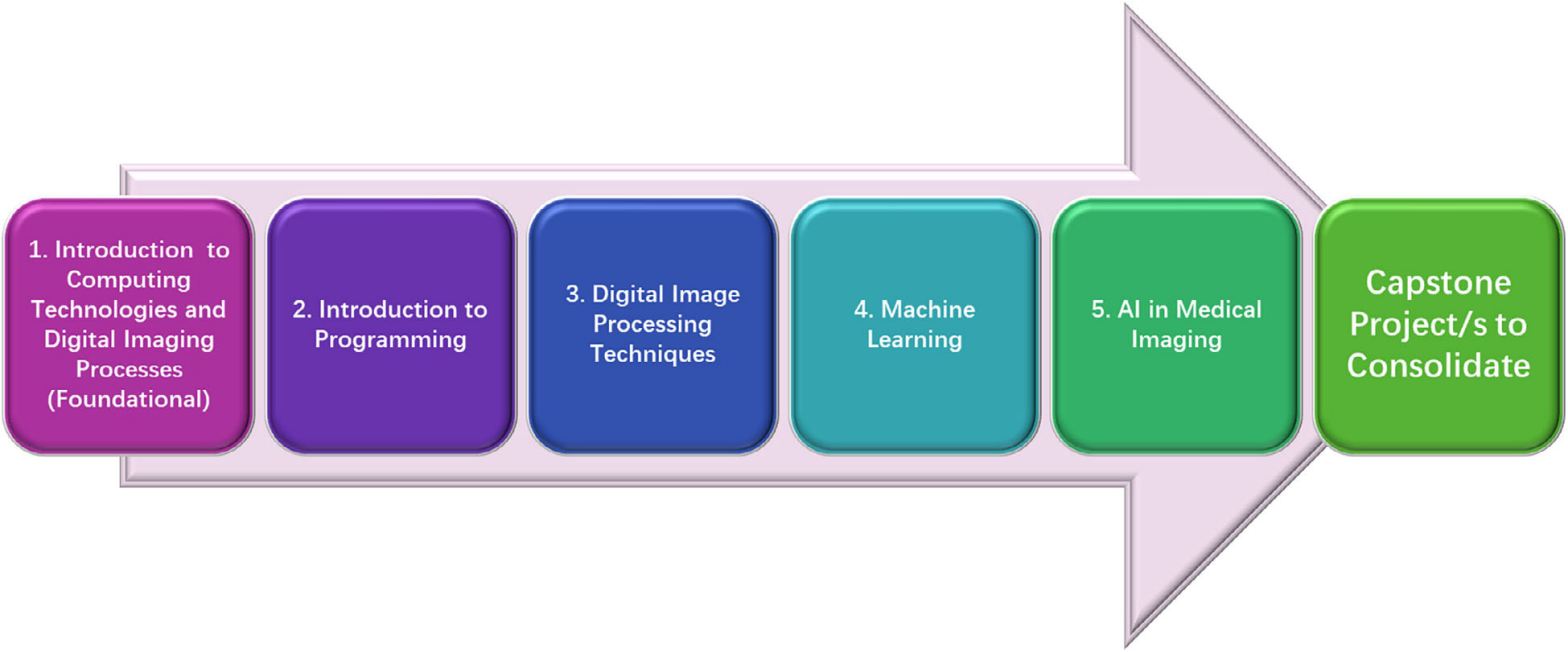
A proposed framework focuses on the curriculum content designed to fast‐track the integration of AI in the training of MITs. AI, artificial intelligence; MITs, medical imaging technologists.

The incorporation of computing technologies into the MI curriculum is essential for preparing students to navigate and excel in an increasingly digital and data‐driven healthcare environment.^30^, ^31^ These technologies encompass a range of skills, including programming, data analysis and ML, all of which are critical for developing and utilising advanced imaging techniques and tools. Recognising the rapid pace of technological advancement, the curriculum framework (Fig. 2) emphasises continuous learning and professional development. Elective courses and continuing education opportunities would enable students and professionals to stay updated on emerging trends, refine their skills and adapt to evolving practices in MI.^32^ This commitment to lifelong learning ensures that graduates remain competitive and capable of driving innovation in the dynamic field of MI.

### Introduction to computing technologies and digital imaging processes (foundational course)

The curriculum is structured to begin with foundational courses that provide students with a solid grounding in the principles of MI while introducing them to essential concepts in computing technologies and digital imaging processing.^33^ ‘Introduction to MI’ could serve as a cornerstone, offering comprehensive insights into various imaging modalities, their underlying principles and their clinical applications.

### Introduction to programming

Programming is fundamental for creating custom imaging algorithms, automating tasks and processing large volumes of imaging data.^34^, ^35^ Introducing students to programming languages commonly used in the MI field, such as Python and MATLAB, can greatly enhance their problem‐solving abilities and technical proficiency. Courses like ‘Introduction to Programming’ can cover the basics of programming languages, including syntax, data structures and control flow, while ‘Applied Programming in MI’ can focus on the development of imaging algorithms, image processing techniques and the automation of routine tasks using programming.^36^

As students evolve, the curriculum advances into specialised areas with courses focusing on cutting‐edge imaging techniques and digital processing methods. ‘Advanced Imaging Modalities’ could explore emerging technologies in MI, keeping students abreast of the latest advancements.

### Digital image processing techniques

Digital image processing techniques could delve into advanced techniques such as image enhancement, segmentation and three‐dimensional reconstruction.^37^ Through practical sessions and lab work, students could gain hands‐on experience working with real‐world imaging datasets, refining their analytical skills and proficiency in manipulating digital images to extract clinically relevant information. Further, data analysis techniques could be included, and these skills are crucial for interpreting large datasets, conducting research and improving imaging protocols.^38^ Training students in data analysis techniques enables them to manage and analyse complex imaging data effectively. A course like ‘Introduction to Data Analysis’ can cover fundamental concepts of data analysis, including statistical methods and data visualisation. An advanced course, ‘Data Analysis in MI’, can apply these techniques to MI datasets, focusing on pattern recognition and trend analysis, which are essential for making informed clinical decisions and advancing research.

### ML and AI in MI

ML, particularly DL, has shown significant potential in improving image analysis and interpretation.^39^, ^40^, ^41^, ^42^ The integration of AI and ML represents a pivotal aspect of the curriculum, reflecting the growing importance of these technologies in MI.^33^ ‘ML Fundamentals’ could introduce students to foundational concepts such as supervised and unsupervised learning, model evaluation, common algorithms and their applications in MI tasks. Equipping students with the knowledge and skills to develop and implement ML models can significantly enhance their ability to leverage AI in clinical practice. Subsequently, a more specialised course ‘AI in MI’ could focus on practical applications, including image classification, segmentation and anomaly detection. This prepares students to leverage AI‐driven tools effectively, enhancing diagnostic accuracy and efficiency in clinical settings.

### Capstone projects to consolidate

Central to the curriculum are practical applications and capstone projects designed to consolidate theoretical knowledge with hands‐on experience.^43^, ^44^ Collaborative capstone projects would provide opportunities for students to tackle complex challenges in MI, integrating computing technologies, digital image processing and AI to propose innovative solutions. Capstone projects, for instance, can involve collaborative efforts to address specific challenges in MI using programming, data analysis and ML. Capstone projects are projects designed to solve real‐world problems or issues that require students to apply knowledge and skills acquired throughout their academic careers. They can be completed individually or as a group in the form of internships, case studies, creative works, research papers and field placement projects.^45^, ^46^

Capstone projects can be in the form of research papers, case studies, creative works, internships and field placement projects. By their nature, they create an opportunity for comprehensive and interdisciplinary engagements that enhance students' critical thinking and problem‐solving skills and afford them the ability to demonstrate their readiness for work in their field.^45^ Additionally, internships and industry partnerships could offer invaluable practical training, exposing students to real‐world scenarios and fostering professional development essential for successful careers in MI.^47^

## Implementation Strategies and Considerations

**Figure 3:**
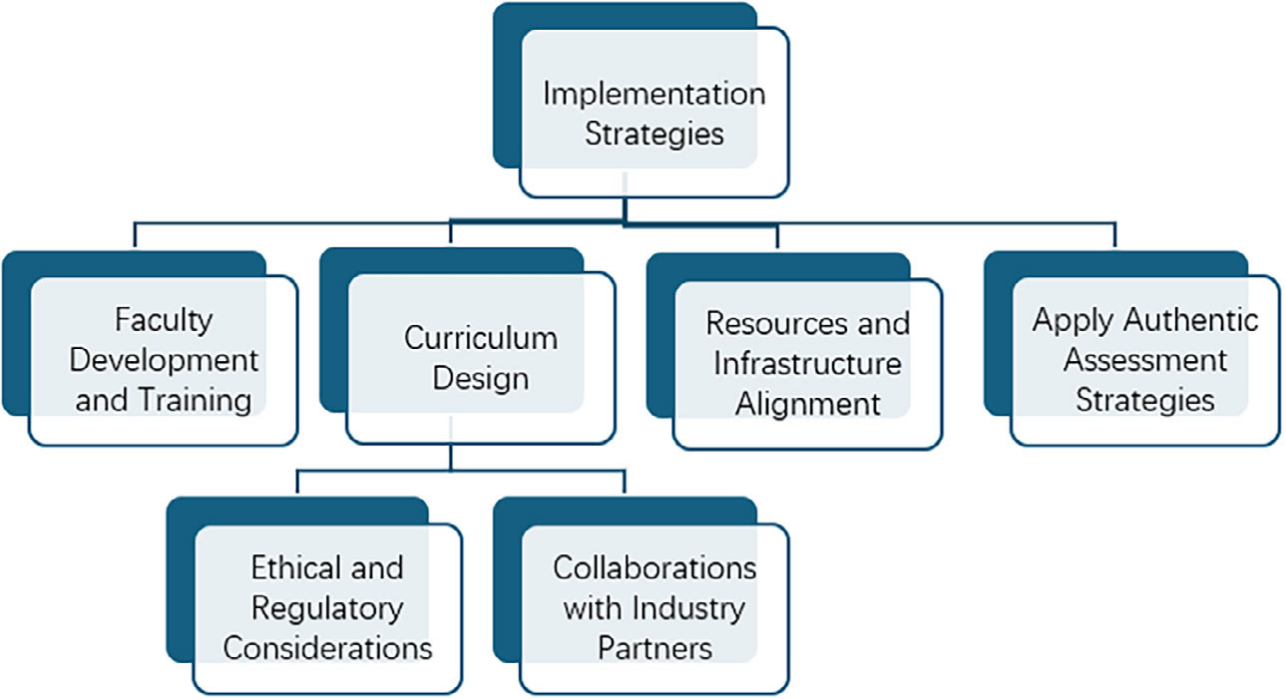
Key implementation strategies and considerations to ensure the successful adoption and effectiveness of the proposed curriculum.

Implementing a comprehensive curriculum framework that integrates computing technologies, digital imaging processing and AI into MI education requires careful planning and consideration of various strategies and challenges. Here, key implementation strategies and considerations are discussed to ensure the successful adoption and effectiveness of the proposed curriculum (Fig. 3).

### Faculty development and training

Central to the successful implementation of the curriculum is the prioritisation of faculty development and training. Ensuring that faculty members are proficient in computing technologies, digital image processing techniques and AI is essential. Establishing robust faculty development programs that encompass workshops, seminars and collaborative learning opportunities can foster ongoing professional growth. These initiatives empower educators to remain current with technological advancements and best practices in MI education. By equipping faculty with the necessary skills and knowledge, institutions can enhance teaching effectiveness and ensure that students receive comprehensive and up‐to‐date instruction.

### Curriculum design

The curriculum design plays a pivotal role in facilitating the seamless integration of foundational and advanced courses in computing, digital image processing and AI. The curriculum design could be aligned with the framework proposed in the previous section. Collaborative efforts between MI specialists, computer scientists and educators will be crucial in maintaining curriculum relevance and alignment with industry standards and clinical practice, fostering an interdisciplinary learning environment that prepares students for diverse challenges in the field:

- Ethical and regulatory considerations are integrated throughout the curriculum to cultivate ethical awareness and responsible conduct among students.^48^ Courses addressing ethical issues, patient privacy, data security and regulatory compliance equip graduates with the knowledge and skills necessary for ethical decision‐making in MI practice. By instilling a strong ethical foundation, institutions prepare students to uphold ethical standards and legal requirements, ensuring their contributions to patient care align with professional and societal expectations.
- Collaboration with industry partners, healthcare institutions and professional organisations enriches the educational experience and enhances career readiness. Industry partnerships provide valuable insights into current practices and technological advancements, offering internship opportunities and mentorship by industry experts.^49^ Collaborative research projects foster innovation in MI technologies, allowing students to contribute to advancements that benefit both the healthcare community and society at large. By fostering interdisciplinary collaboration and industry engagement, educational institutions prepare students to thrive in dynamic healthcare environments, where innovation and collaboration are the key drivers of progress.

### Resources and infrastructure alignment

Access to state‐of‐the‐art resources and infrastructure is imperative for enriching the educational experience and facilitating hands‐on learning. Providing students with access to specialised software, high‐performance computing facilities and advanced imaging equipment allows for practical applications and research opportunities. Dedicated laboratories equipped with the latest technology enable students to manipulate imaging datasets and experiment with computational tools, thereby honing their skills in digital image processing and AI applications.^40^ Partnerships with industry leaders and healthcare institutions further enhance resource accessibility, offering students exposure to real‐world datasets, collaborative research projects, and professional networking opportunities that bolster their readiness for careers in MI. Further, partnering with industry creates opportunities to secure adequate funding and resources, which is essential for integrating advanced technologies into MI education.

### Application of authentic assessment strategies

Effective and authentic assessment strategies are essential for evaluating student learning outcomes and the overall effectiveness of the curriculum.^50^ Practical assignments, project‐based assessments and performance evaluations that measure proficiency in computing technologies, digital imaging processing techniques and AI algorithms provide valuable insights into student progress. Continuous feedback from students, faculty and industry partners ensures that assessment methods remain responsive to evolving educational and professional needs, guiding curriculum enhancements and refinements that optimise learning outcomes.

## Recommendations for Future Research

Future research is essential to evaluate the effectiveness of the proposed curriculum framework in real‐world educational settings. Studies should focus on how well this framework prepares students for the challenges they will face in MI practice. Additionally, exploring emerging technological advancements that can be integrated into MI education is crucial to ensure curricula remain relevant and responsive to the dynamic nature of the field. This focus on continuous improvement will help align educational practices with the evolving landscape of MI technologies, ultimately enhancing the training and readiness of future professionals.

## Conclusion

A comprehensive curriculum framework that integrates computing technologies, digital image processing and AI into MI education is proposed in this paper, aiming to prepare future professionals to navigate and innovate within this dynamic landscape. Integrating computing technologies and AI into MI education not only enhances diagnostic capabilities and patient care but also equips future professionals with the skills needed to thrive in an increasingly digital healthcare environment. Through strategic curriculum design, faculty development and ethical education, educational institutions can empower the next generation of MI specialists to lead advancements that improve health outcomes and shape the future of healthcare.

## Conflict of Interest

The author declares that he has no financial or personal relationship(s) that may have inappropriately influenced him in writing this article.

## Data Availability

Data sharing is not applicable to this article as no new data were created or analyzed in this study.
